# Green tea intake and its effect on laboratory parameters and disease symptoms in hospitalised patients with Covid 19: a structured protocol for a randomized controlled trial

**DOI:** 10.1186/s13063-021-05462-8

**Published:** 2021-08-03

**Authors:** Sara Mahmoodi, Mojtaba Yousefi, Omid Sadeghi, Ali Mahmoodabadi, Mohammadreza Sadriirani, Zahra Hosseinzade, Abdolhossein Jahanbakhsh, Seyed Bahman Panahande, Zaker Saeedinejad, Jan Mohamad Malekzadeh, Mohsen Naghmachi, Azizollah Pourmahmoudi

**Affiliations:** 1 Department of Nutrition, School of Health and nutrition, Yasuj University of Medical Sciences, Yasuj, Iran; 2 Department of Community Nutrition, School of Nutritional Sciences and Dietetics, Tehran University of Medical Sciences, Tehran, Iran; 3 Assistant Professor of Nutrition, Department of Nutrition, School of Health and nutrition, Yasuj University of Medical Sciences, Yasuj, Iran; 4 Assistant Professor of Infectious Disease, Department of Internal Medicine, School of Medicine, Yasuj University of Medical Sciences, Yasuj, Iran; 5 Instructor of Biology, Department of Nutrition, School of Health and Nutrition, Yasuj University of Medical Sciences, Yasuj, Iran

**Keywords:** COVID-19, Randomised controlled trial, protocol, ESR, CRP, CBC, green tea supplement

## Abstract

**Objectives:**

The current randomized controlled trial (RCT) will be conducted to assess the effect of green tea intake on disease symptoms and laboratory parameters including C-reactive protein (CRP), erythrocyte sedimentation rate (ESR), and complete blood count (CBC) in patients with mild-to-moderate Covid-19 infection.

**Trial design:**

Randomized, double-blinded, parallel (1:1 ratio) clinical trial exploratory study

**Participants:**

We will recruit patients with COVID-19 infection admitted to Yasuj Shahid Jalil Hospital in Yasuj City, Kohgiluyeh and Boyer-Ahmad Province, Iran.

Participants’ inclusion criteria are as follows:

- Inclusion Criteria
  - Patients aged ≥18 years
  - COVID-19 diagnosis according to real-time polymerase chain reaction (RT-PCR)
- Exclusion Criteria
  - Pregnancy or lactation
  - Disseminated intravascular coagulation or any other types of coagulopathy
  - Severe congestive kidney failure
  - Having a history of participating in a clinical trial during the last 30 days

**Intervention and comparator:**

**Intervention**: Two capsules containing 450 mg green tea extract along with routine treatment for COVID-19 patients in the intervention group. Two capsules containing placebo plus routine treatment for patients with COVID-19 infection. Capsules will be taken twice a day, after lunch and dinner, for 14 days.

**Main outcomes:**

Changes in disease symptoms and laboratory parameters including CRP, ESR, and CBC after 14 days of the intervention compared to control group.

**Randomisation:**

Eligible patients will be randomly assigned into the intervention or control group in a 1:1 ratio. Randomization will be performed based on 8 permuted blocks with block sizes of 10, and patients in the intervention and control groups will be matched according to sex and age categories. Randomization will be done using computer-generated random numbers (Randomization.com)

**Blinding (masking):**

The appearance of placebo and green tea capsules will be similar in terms of shape and color, and they will be packed in the same bags that will be prepared by the company. Also, the researcher and all participants will not be aware of the divisions until the end of the study.

**Numbers to be randomised (sample size):**

The total sample was determined based on CRP MCID in which high CRP levels were considered >2.6 mg/L. Accordingly, a total sample size of 37 patients for each intervention group was required.

**Trial Status:**

The protocol is Version 1.0, on June 5, 2021. Recruitment will start on July 11, 2021, which is anticipated to be completed by September 21, 2021.

**Trial registration:**

IRCT20150711023153N3 (https://www.irct.ir/trial/55948) retrospectively registered on June 4, 2021

**Full protocol:**

The full protocol is attached as an additional file, accessible from the Trials website (Additional file 1). In the interest in expediting dissemination of this material, the familiar formatting was eliminated; this Letter serves as a summary of the key elements of the full protocol.

**Supplementary Information:**

The online version contains supplementary material available at 10.1186/s13063-021-05462-8.

## Supplementary Information

**Additional file 1.** Full study protocol.

## Data Availability

The final dataset of trial will be available upon request from the primary investigator via e-mail at panahande.b@gmail.com, after obtaining permission from Regional Ethics Committee

