# Supplementary material for: Green tea intake and its effect on laboratory parameters and disease symptoms in hospitalised patients with Covid 19: a structured protocol for a randomized controlled trial
Source: Trials. 2021 Aug 3;22:514. doi: 10.1186/s13063-021-05462-8 (PMC8329408; doi:10.1186/s13063-021-05462-8)
Supplement: Supplementary file 1 — Additional file 1. Full study protocol. [file 13063_2021_5462_MOESM1_ESM.docx]

**Evaluation of the effect of green tea supplement on inflammatory biomarkers, CRP, ESR and CBC in patients with Covid 19 patients in shahid beheshti hospital in yasuj**

**Backgrounds and Objectives:**

Corona viruses A wide range of viruses that cause sickness in mammals and birds. In humans, they cause sicknesses from colds to respiratory syndrome coronavirus 2 (SARS-CoV2) [1-2]. Also, in the (sars-cov2) disease, which was announced by the World Health Organization, Covid19 virus, the 7th generation of Corona viruses has a role to play. This is a RNA positive, single string, Beta cell kind of viruses [5-3]. Also, these viruses have a very high mutagenic rate, and this is very effective in the severity of pathogenicity and mortality in this disease [6-8]. The severity of symptoms is individualized and varies from mild to inflammatory and fatal conditions. The mild type of this disease is known as the absence of pneumonia [9], but severe type respiratory, hypoxia and organ failure are more pronounced in severe and fatal types [3]. And also severity of this disease has several factors such as viral dose and existence of underlying diseases in individuals, the important ones are Diabetes, blood pressure, chronic pulmonary obstruction, heart disease and kidney disease [3].

The Commune period of the disease is 2 to 14 days, with a longer commune period than other respiratory diseases such as influenza, which causes more people in society [1]. One of the main symptoms of this disease mainly involves fever, headache, runny nose, sore throat and difficulty in breathing [5].

The main mechanism in the transfer of this disease is person to person through respiratory droplets or Virus-infected hand contact to the nasal mucus or healthy person eyes. There are several ways to diagnose the disease in individuals, which PCR test is the most important way to diagnose as a confirmation method in this disease. Another way, including blood tests, is also used to diagnose this disease, and the virus is also recognizable in the feces of infected people [1-3-4-10-12].

The main mechanism of predicted pathogenicity for this disease is the binding of the virus to Angiotensin receptors 2 That causes the involvement of T type white blood cell and thus increasing inflammatory factors and creating cytokine storm and stimulation of inflammatory cells of Interleukin 6 and 10 [2-3]

By January 15, 2021, the virus has caught more than 93 million patients in the world, and has registered more than 2.5 million deaths, the disease in our Iran has also caught 1 million and 350 thousand patients and 56 thousand and 300 people have recorded death [13].

This disease does not have a definite drug, but many specialists use drugs such as chloroquine, Hydroxy chloroquine, e-naproxen and a combination of these cases, but the effect of these drugs has not yet been proven. The issue is while strengthening the immune system can prevent inflammatory processes and inhibit cytokine storm and reduces the severity of the symptoms of the disease and will accelerate the recovery process, including immune boosting food that can contribute to this such as green tea. [2-4-11-12-14]

Green tea is produced from the leaf of Camellia sinensis, which has several compounds, including catechin, which depends on the season, weather, gardening methods and plant age [38]. The species of catechins in green tea include: Epigallocatechin gallate (EGCG), epicatechin gallate (ECG), epicatechin (EC), gallocatechin gallate (GCG). Older plants have more polyphenol catechins[38]. Of these four cases, EGCG has the highest concentration among catechins in green tea, which has several effects including anticancer, anti-inflammatory, anti-microbial and antioxidant (role in regulating vital signs) and its benefits in cardiovascular diseases and the effects of oral and teeth [15-16]. Also, based on studies of GTCs (green tea catechins), especially Epigallocatechin gallate (EGCG), have shown antiviral effects against diverse viruses [17].

A molecular study showed that EGCG, with a strong binding energy to the main protease inhibitors (MPRO), which plays a role in creating molecules of the virus, is a very good phytochemical ability to inhibit glycoprotein S and the main protease of the covid 19 virus (MPRO). According to the results of this study, phenolic compounds have the right inhibition for MPRO protease and S virus protein in comparison with chemical drugs [18-5].

Green tea can change the infection of influenza virus by interacting with viral hemagglutinin (HA) and with the synthesis of viral RNA in cells, in addition, catechins inhibit the activity of indonuclease of RNA polymerase viral.

Therefore, the potential use of green tea and its constructive catechins to protect against influenza, which is from viral diseases, attracted attentions [19]. And various studies such as Shin and colleagues in 2020 have shown this plant in vitro on SARS-COV-2 so that Epigallocatechin gallate (EGCG) significantly disable SARS-COV-2 [20]. Also, in the study of Chu and colleagues in 2018, green tea consumption was accompanied more than 2 times a day with a decrease in the risk of COPD in the Korean population [21], as well as according to Baso and colleagues in 2013, green tea extract significantly increased plasma antioxidant capacity and glutathione of blood completed [22, 23]

There are very limited data on the drug interactions of green tea and their components in humans, according to animal studies, green tea or major secondary metabolites, should be used with alert in people with liver disease and in combination with narrow therapeutic indexes. Also, the absorption of these compounds after meals is reduced due to reduced bioavailability, and the plant also reduces the effects of warfarin drug anticoagulants, and these people are better not to use this drug based on the papers of green tea toxocytes [38].

According to what was said and due to the global epidemic of this disease and extensive costs in the treatment of this disease and the lack of a definite drug in the treatment process on the one hand, as well as the recognition of the pathophysiology of the disease that can be through MPRO and on the other hand. Considering what is said about green tea properties, it can be suggested that prescribing can have positive effects on improving or decreasing the progression of the disease. Therefore, the study of green tea on inflammatory factors in patients with patients seems necessary.

**Material and Methods:**

**Participants & recruitment:**

Out of the total number of hospitalized patients, 48 ​​patients with definitive diagnosis based on PCR test, who also have other inclusion criteria, are included in the study by availability sampling.

**Study Design & randomization:**

In this research, after reviewing the research background and review of articles and books on the effect of nutrition in the recovery of inflammatory diseases, infectious, pulmonary and immune enhancement, research goals were determined. Then, identifying and selecting samples. The present study will be conducted as a randomized double-blind randomized clinical trial in patients with Covid 19 hospitalized in Yasuj Shahid Beheshti Hospital.

Based on the Stata software and MCID>2.6 with a 95% confidence coefficient and the failure coefficient of 80%, according to Nimin and colleagues, the sample size of individuals in each intervention group and placebo will be 37 people, a total of 74 people in the study [35,40].

Of them, those who wish to cooperate in this project, with direct researcher request, 74 patients with Covid 19 with a definite diagnosis based on the PCR test, are admitted to the hospital will be invited to the study by Inclusion criteria and Non- Inclusion criteria, then the purpose of the research is explained for the patient and his/her companions, and according to Inclusion criteria, the subjects are investigated in the process of research.

Patients will be Stratify by Block Random sampling based on age and sex in one of 8 blocks of 9 and 10 people and by computer generated random numbers (Randomization.com) in one of two intervention or placebo groups, this list is in the hands of the group's secretary and Who Does not play a role in the study

Blinding of patients, health care professionals, principal investigators as well as data collectors and data analysts will be unaware of the intervention and placebo groups still the end of the study. The intervention includes a placebo or green tea extract capsule in special package and exactly same in color and taste are labeled with different numbers, and the contents of these numbers will be kept in a closed envelope in the faculty and its contents will be preserved until the end of the study.

After obtaining written consent from patients, public questionnaires included demographic and anthropometric data, and 24-hour feed questionnaire (one in the hospital and at home if the patient's discharge) by experienced experts with interview method of Face to face or telephone is recorded from the patient or the closest person to the patient and the consumption of supplements and drugs is recorded by experienced experts in the interview method.

Then, these patients who hasn’t eat any food since they woke up will give 10 cc blood to be evaluated for three blood invoices. After that, patients were randomly assigned in two groups receiving green tea (with 450 mg of each capsule and 900 mg / day) twice a day after lunch and the other after dinner and intervention for 14 days Until the end of the recovery and discharge from the hospital, before the time of discharge from the section or on the same day, the tests and measurements will be re-implemented and recorded again.

Meanwhile, at the end of the 14-day period, the quarantine completion will be followed up by a researcher in order to consume appropriate amounts and reminders necessary for blood sampling. The length of treatment (hospitalization days), hospitalization in the intensive care unit, the use of precise dose therapy drugs and patients' mortality and patient mortality rates daily by the responsible expert and examined, also recorded information about Plan is made by one of the researchers

At the end of the data, SPSS software version 21 entered. Finally, the results are analyzed and interpreted. Bloods will also be handed over to the hospital and delivered to the laboratory, placebo or green tea will be given to patients for 14 days and if discharged The hospital will be delivered daily on their home daily, and will be followed up of consumption, patients referring to home with guidance and follow-up on day 14 as fasting to the laboratory and will be blooded and if able to Referral to the lab, on the day before the day 14, this item will be identified and with one nurse will be blooded to the patient for blood sampling, if the patient will continue to study for any reason to continue to study on the same day. Next follow-ups to determine the effect of confounds and minimize their effect, as well as to accurately implement the intervention and participating in the post of 14 days of being discharged from the hospital. Executive work will be conducted by a sector nurse with Dr. Saeedi's supervision of infectious specialists in the hospital, as well as surveillance and rebellion of 3 students as a researcher in the plan

**Study eligibility:**

***Inclusion criteria:***

(1) informed consent

(2) at least 18 years of age

(3) diagnosis of Covid-19 based on the polymerase chain reaction test (PCR)

(4) tendency to participate in research study

***Non-inclusion criteria***

(1) pregnancy or lactation

(2) Increase a common disease that can increase the risk of thrombosis.

(3) Released intra-vascular coagulation

(4) extreme congestive heart failure [39-34] To diagnose a doctor

(5) other contraindications that are determined by patient physicians

(6) participate in any antiviral clinical trial Covid-19 within the past 30 days, before signing up in the green tea plan.

***Exclusion criteria***

(1) severe septic shock

(2) Other non-denying other green tea used by patient physicians

(3) patient death

(4) patient's unwillingness to continue presence in the plan

**Clinical, Para clinical and dietary intake assessments:**

A demographic questionnaire containing information about age, sex, habitat, education, occupation etc., will be taken from patients. Medical history and medications/supplements history will also be asked of the patient or his companion.

Height, weight and waist to hip ratio (WHR) will be measured by standard methods [4[1](#_ENREF_1)] as well as the body mass index (division of weight in kilograms to the height in meters squared).

For the purpose of evaluating detailed information about nutrient intake and the food pattern, a 24-hour dietary recall for 3 days (including a weekend day) will be taken by a well-trained interviewer. Dietary intakes will be entered into Nutritionist IV software for further analysis.

10 cc blood samples will be obtained after 12 hour overnight fasting to measure complete blood count (CBC) and inflammatory indices including CRP, ESR, at the baseline and by the end of week-2. In the case of patient discharge earlier than scheduled, they will be followed up after discharge and receive Green Tea or placebo. The length of hospital stay, disease complications, drug and prescription dosing and mortality rate will be recorded on a daily basis. If the patient refuses to continue the study for any reason, blood sample will be drawn on the same day.

**Follow up:**

Subsequent follow-up is to detect and minimize the effects of the confounding factors, as well as to accurately carry out the intervention protocol and will be done by well-trained, committed researchers.

**Study objectives:**

***Primary objectives***

Determine the effect of green tea on inflammatory biomarkers -CRP, ESR and CBC in patients with Covid 19 hospitalized in Shahid Beheshti Hospital of Yasuj

***Secondary objectives***

1- Determine and compare the average serum concentration of CRP before and after intervention and between the intervention group with medication

2. Determine and compare the average serum concentration of ESR before and after the intervention and between the intervention group with medication

3- Determine and compare the average serum concentration of CBC factors before and after intervention and between intervention group with medication

**Data analysis:**

The data will be analyzed by SPSS software version 21. After examining the normality distribution of variables using Kolmogorov-Smirnov test, quantitative normal variables will be reported as mean±standard deviation, quantitative non-normal variables will be reported as median IQR, and qualitative variables as number (percentage). Independent T Test and Mann Whitney test will be used to compare the mean of variables between two groups. Paired T test or Wilcoxon test will be used to analyses within groups means. P-Value <0.05 will be considered as statistically significant. If the patient refuses to continue the study for any reason, statistical methods (Intention-to-treat analysis) will be performed.

1. Kakodkar, P., N. Kaka, and M. Baig, *A comprehensive literature review on the clinical presentation, and management of the pandemic coronavirus disease 2019 (COVID-19).* Cureus, 2020. **12**(4).

2. Guo, Y.-R., et al., *The origin, transmission and clinical therapies on coronavirus disease 2019 (COVID-19) outbreak–an update on the status.* Military Medical Research, 2020. **7**(1): p. 1-10.

3. He, F., Y. Deng, and W. Li, *Coronavirus disease 2019: What we know?* Journal of medical virology, 2020. **92**(7): p. 719-725.

4. Chawla, D., et al., *Perinatal-neonatal management of COVID-19 infection—guidelines of the Federation of Obstetric and Gynaecological Societies of India (FOGSI), National Neonatology Forum of India (NNF), and Indian Academy of Pediatrics (IAP).* Indian pediatrics, 2020. **57**(6): p. 536-548.

5. Ghosh, R., et al., *Evaluation of green tea polyphenols as novel corona virus (SARS CoV-2) main protease (Mpro) inhibitors–an in silico docking and molecular dynamics simulation study.* Journal of Biomolecular Structure and Dynamics, 2020: p. 1-13.

6. Wu, Z. and J.M. McGoogan, *Characteristics of and important lessons from the coronavirus disease 2019 (COVID-19) outbreak in China: summary of a report of 72 314 cases from the Chinese Center for Disease Control and Prevention.* Jama, 2020. **323**(13): p. 1239-1242.

7. Jovic, T.H., et al., *Could vitamins help in the fight against COVID-19?* Nutrients, 2020. **12**(9): p. 2550.

8. Nachega, J., M. Seydi, and A. Zumla, *The late arrival of coronavirus disease 2019 (Covid-19) in Africa: mitigating pan-continental spread.* Clinical Infectious Diseases, 2020. **71**(15): p. 875-878.

9. Asgary, S., et al., *Clinical evaluation of blood pressure lowering, endothelial function improving, hypolipidemic and anti‐inflammatory effects of pomegranate juice in hypertensive subjects.* Phytotherapy Research, 2014. **28**(2): p. 193-199.

10. Kalantar-Zadeh, K. and L.W. Moore, *Impact of nutrition and diet on COVID-19 infection and implications for kidney health and kidney disease management.* Journal of renal nutrition, 2020. **30**(3): p. 179-181.

11. Yoo, J.-W., et al., *Clinical impact of supplementation of vitamins B1 and C on patients with sepsis-related acute respiratory distress syndrome.* Tuberculosis and Respiratory Diseases, 2020. **83**(3): p. 248.

12. Horowitz, R.I. and P.R. Freeman, *Three novel prevention, diagnostic, and treatment options for COVID-19 urgently necessitating controlled randomized trials.* Medical hypotheses, 2020. **143**: p. 109851.

13. who.

14. Zabetakis, I., et al., *COVID-19: the inflammation link and the role of nutrition in potential mitigation.* Nutrients, 2020. **12**(5): p. 1466.

15. Reygaert, W.C., *An update on the health benefits of green tea.* Beverages, 2017. **3**(1): p. 6.

16. Oz, H.S., *Chronic inflammatory diseases and green tea polyphenols.* Nutrients, 2017. **9**(6): p. 561.

17. Xu, J., Z. Xu, and W. Zheng, *A review of the antiviral role of green tea catechins.* Molecules, 2017. **22**(8): p. 1337.

18. Poy, D., M. Tohidfar, and M.S. Nasrollahzadeh, *Investigate the inhibitory effects of plant secondary metabolites Compared with standard drugs on Mpro protease and spike glycoprotein SARS-CoV-2 by Molecular Docking.* Journal of Cellular and Molecular Research (Iranian Journal of Biology), 2020. **33**(4): p. 484-498.

19. Lee, H., et al., *Anti-influenza virus activity of green tea by-products in vitro and efficacy against influenza virus infection in chickens.* Poultry science, 2012. **91**(1): p. 66-73.

20. Ohgitani, E., et al., *Significant inactivation of SARS-CoV-2 by a green tea catechin, a catechin-derivative and galloylated theaflavins in vitro.* BioRxiv, 2020.

21. Oh, C.-M., et al., *Consuming green tea at least twice each day is associated with reduced odds of chronic obstructive lung disease in middle-aged and older Korean adults.* The Journal of nutrition, 2018. **148**(1): p. 70-76.

22. Serban, C., et al., *Effects of supplementation with green tea catechins on plasma C-reactive protein concentrations: A systematic review and meta-analysis of randomized controlled trials.* Nutrition, 2015. **31**(9): p. 1061-1071.

23. Basu, A., et al., *Green tea supplementation increases glutathione and plasma antioxidant capacity in adults with the metabolic syndrome.* Nutrition Research, 2013. **33**(3): p. 180-187.

24. Li, L., et al., *Metabolic impact of weight loss induced reduction of adipose ACE-2–Potential implication in COVID-19 infections?* Metabolism, 2020. **113**: p. 154401.

25. Beigel, J.H., et al., *Remdesivir for the treatment of Covid-19—preliminary report.* The New England journal of medicine, 2020.

26. Boulware, D.R., et al., *A randomized trial of hydroxychloroquine as postexposure prophylaxis for Covid-19.* New England Journal of Medicine, 2020. **383**(6): p. 517-525.

27. Esmaeilinezhad, Z., et al., *The effect of synbiotics pomegranate juice on cardiovascular risk factors in PCOS patients: a randomized, triple-blinded, controlled trial.* Journal of endocrinological investigation, 2020. **43**(4): p. 539-548.

28. Furushima, D., et al., *Prevention of acute upper respiratory infections by consumption of catechins in healthcare workers: a randomized, placebo-controlled trial.* Nutrients, 2020. **12**(1): p. 4.

29. Bagheri, R., et al., *Does green tea extract enhance the anti‐inflammatory effects of exercise on fat loss?* British journal of clinical pharmacology, 2020. **86**(4): p. 753-762.

30. پوی, et al., *بررسی مهارپذیری متابولیت های ثانویه گیاهی در مقایسه با داروهای شیمیایی بر روی پروتئاز اصلی Mpro و Spike گلیکوپروتئین ویروس SARS-CoV-2 (nCov-19) به روش داکینگ مولکولی.* مجله پژوهش‌های سلولی و مولکولی (مجله زیست شناسی ایران)(علمی), 2020. **33**(4): p. 484-498.

31. Lowe, G.M., K. Gana, and K. Rahman, *Dietary supplementation with green tea extract promotes enhanced human leukocyte activity.* Journal of Complementary and Integrative Medicine, 2015. **12**(4): p. 277-282.

32. Eichenberger P, Mettler S, Arnold M, Colombani PC. No effects of three-week consumption of a green tea extract on time trial performance in endurance-trained men. International journal for vitamin and nutrition research. 2010 Jan 1;80(1):54.

33. cdc.

34. Hunt, R.H., et al., *COVID-19 and Gastrointestinal Disease: Implications for the Gastroenterologist.* Digestive Diseases, 2020.

35. Lee, S.M. and W.S. An, *New clinical criteria for septic shock: serum lactate level as new emerging vital sign.* Journal of thoracic disease, 2016. **8**(7): p. 1388.

36. Hassaniazad, M., et al., *The clinical effect of Nano micelles containing curcumin as a therapeutic supplement in patients with COVID-19 and the immune responses balance changes following treatment: A structured summary of a study protocol for a randomised controlled trial.* Trials, 2020. **21**(1): p. 1-3.

37. Safa, O., et al., *Effects of Ginger on clinical manifestations and paraclinical features of patients with Severe Acute Respiratory Syndrome due to COVID-19: A structured summary of a study protocol for a randomized controlled trial.* Trials, 2020. **21**(1): p. 1-2.

38 Bedrood, Zeinab, Maryam Rameshrad, and Hossein Hosseinzadeh. "Toxicological effects of Camellia sinensis (green tea): A review." *Phytotherapy Research* 32.7 (2018): 1163-1180.‏

39 cdc

40 Nieman DC, Henson DA, Maxwell KR, Williams AS, McAnulty SR, Jin F, Shanely RA, Lines TC. Effects of quercetin and EGCG on mitochondrial biogenesis and immunity. Medicine & Science in Sports & Exercise. 2009 Jul 1;41(7):1467-75..

41 He, F., Y. Deng, and W. Li, Coronavirus disease 2019: What we know? Journal of medical virology, 2020. 92(7): p. 719-725
